# A review of the genus *Takecallis* Mastumura in Korea with the description of a new species (Hemiptera, Aphididae)

**DOI:** 10.3897/zookeys.748.23140

**Published:** 2018-04-05

**Authors:** Yerim Lee, Seunghwan Lee

**Affiliations:** 1 Laboratory of Insect Biosystematics, Department of Agricultural Biotechnology, Research Institute of Agriculture and Life Sciences, Seoul National University, Seoul 08826, Republic of Korea

**Keywords:** Aphid, Bamboo pest, Calaphidinae, COI, Panaphidini

## Abstract

The aphid genus, *Takecallis* Mastumura, 1917, was reviewed from Korea. Four species, *T. alba* Y. Lee, **sp. n.**, *T. arundicolens* (Clarke), *T. arundinariae* (Essig), and *T. taiwana* (Takahashi), are recognized in Korea and morphological and molecular evidence are presented. Species descriptions and illustrations are given for the four species. A key to Korean species and the results of COI sequence analyses are also provided.

## Introduction

The genus *Takecallis* was established by Matsumura (1917) based on the type species *T. arundicolens*. This is one of the small aphid genera of the tribe Panaphidini (Aphididae: Calaphidinae). In this genus, six species are known around the world (Remaudière and Remaudière 1997, Favret 2017). All known species have been described from Southeast Asian countries such as China (Qiao and Zhang 2004), India (Chakrabarti 1988), Japan (Higuchi 1968), Korea (Quednau and Lee 2001), and Taiwan (Quednau 2003). However, some species such as *T. arundicolens*, *T. arundinariae*, and *T. taiwana* were introduced into Australia (Valenzuela et al. 2010), England (Higuchi 1968), Hungary (Basky and Neményi 2014), Netherland (Pion 2009), New Zealand (Higuchi 1968), North and South America (Delfino 2001, Foottit et al. 2006, Gonzáles et al. 2000, Halbert et al. 2000, Lazzari et al. 1999, Simbaqueba et al. 2016), and South Africa (Quednau 1962). *Takecallis* species have a monoecious holocyclic life cycle on various bamboos (*Arundinaria* spp., *Bambusa* spp., *Dendrocalamus* spp., *Phyllostachys* spp., *Pleioblastus* spp., *Pseudosasa* spp., *Sasa* spp., and *Yushania* sp.) belonging to the family Poaceae (Qiao and Zhang 2004, Quednau 2003).

In Korea, three species, *Takecallis arundicolens*, *T. arundinariae*, and *T. taiwana*, have so far been recorded in this genus (Quednau and Lee 2001). However, recent DNA barcoding revealed that there is an undescribed species in Korea (Lee et al. 2017). In this study, a large number of *Takecallis* samples were collected in Korea and examined together with museum specimens. We also conducted molecular analyses based on the partial mitochondrial cytochrome oxidase subunit I (COI) of fresh-ethanol preserved samples. Generally, COI barcoding provides a good enough resolution for species identification in aphids (Foottit et al. 2008, Lee et al. 2011).

A total of four species were recognized in Korea. Both morphological and molecular evidence strongly indicated that *T. alba* Y. Lee, sp. n. is a valid species. Here, together with a description of the new species, photographs of live aphids and illustrations are provided along with a key to species of the genus *Takecallis* in Korea. Pairwise distance analyses and a neighbor-joining tree based on the partial COI sequence are also provided.

## Materials and methods

Aphid samples were collected in South Korea from 1999 to 2015. All samples were preserved in 90–95% ethanol for over one month, and then mounted in Canada balsam, following the methods of Blackman and Eastop (2000) and Martin (1983). Illustrations for each species were taken with a digital camera attached to the microscope (Leica 400B, Leica Microsystems, Germany) at a resolution of 600 dpi. Measurements for each specimen are taken from the digital images by using image analysis software, Active measure ver. 3.0.3 (Mitani Co. Ltd, Japan). All specimens are deposited in the National Academy of Agricultural Science (NAAS), Jeonju-si, Republic of Korea and the College of Agriculture and Life sciences, Seoul National University, Republic of Korea (CALS SNU).

Aphid samples were identified using keys to *Takecallis* species by Higuchi (1972) and Quednau (2003). For further confirmation, DNA barcoding results were also applied.

Abbreviations used for diagnosis, description, figures, and Table 1 are:

**BL** body length;

**ANT** antennae;

**ANT I-VI** antennal segments I–VI;

**BASE** basal part of last antennal segment;

**PT** processus terminalis of last antennal segment;

**Ls ANT III** longest setae on ANT III;

**BD III** basal diameter of ANT III;

**URS** ultimate rostral segment;

**Co** costa;

**Cu** cubitus;

**M** media;

**Pts** pterostigma;

**Rs** radial sector;

**FEM** hind femur;

**TIB** hind tibiae;

**HT 2** second segment of hind tarsus;

**SIPH** siphunculus;

**ABD TERG I-VIII** abdominal tergites I-VIII.

Provincial names in South Korea for the collection data are abbreviated as follows: CN, Chungcheongnam-do; GB, Gyeongsangbuk-do, GN, Gyeongsangnam-do; GW, Gangwon-do; JJ, Jeju-do; JN, Jeollanam-do.

Main morphological characters such as measurements (in mm), number of setae on antennal segments, number of rhinaria, and body part ratios of Korean *Takecallis* are given in Table 1.

**Table 1.** Biometric data of *Takecallis* species in Korea.

	Body parts	*T. alba* sp. n.	*T. arundicolens*	*T. arundinariae*	*T. taiwana*
		(n = 20)	(n = 20)	(n = 20)	(n = 14
Length (mm)	BL	2.08–2.51	1.57–1.89	1.90–2.65	2.21–2.48
	ANT	3.36–4.00	2.36–2.51	2.54–3.41	1.61–1.88
	ANT I	0.12	0.07–0.09	0.09–0.12	0.08
	ANT II	0.09–0.11	0.07–0.08	0.09–0.12	0.06–0.09
	ANT III	1.07–1.33	0.67–0.72	0.70–1.11	0.57–0.67
	ANT IV	0.73–0.91	0.46–0.51	0.54–0.85	0.31–0.36
	ANT V	0.60–0.69	0.46–0.48	0.48–0.77	0.26–0.33
	BASE	0.34–0.40	0.27–0.31	0.26–0.40	0.15–0.20
	PT	0.34–0.44	0.33–0.36	0.31–0.40	0.17–0.19
	URS	0.05	0.05–0.06	0.05–0.06	0.07
	FEM	0.55–0.69	0.41–0.46	0.53–0.66	0.46–0.50
	TIB	0.87–1.15	0.72–0.80	0.91–1.21	0.79–0.88
	HT 2	0.10–0.11	0.09–0.10	0.10–0.12	0.11–0.13
	SIPH	0.08–0.11	0.04–0.05	0.05–0.08	0.04–0.05
	Cauda	0.12–0.14	0.14–0.15	0.11–0.16	0.15–0.20
	Ls ANT III	0.01–0.02	0.01	0.01	0.01–0.02
No. of setae on	ANT I	5–6	4–5	3–5	3–4
	ANT II	2–3	2–3	2–3	3
	ANT III	24–39	13–23	18–28	17–21
	BASE	1	1	1	1
	URS (accessory setae)	4	4–5	4	4–5
	SIPH	1	1	1	0
	ABD TERG VIII	2	2	2	2
	Cauda knob	9–12	9–13	10–15	12–13
	Each lobe of anal plate	7–10	7–8	8–12	10
No. of rhinaria on	ANT III	4–7	4–7	5–10	5–7
	ANT IV	0	0	0	0
	ANT V	1	1	1	1
Ratio (times)	ANT / BL	1.48–1.70	1.30–1.60	1.22–1.39	0.73–0.79
	PT / BASE	0.89–1.11	1.10–1.26	0.94–1.27	0.90–1.06
	PT / ANT III	0.31–0.34	0.47–0.50	0.35–0.47	3.32–3.72
	URS / HT 2	0.45–0.50	0.56–0.67	0.42–0.60	0.54–0.64
	URS / BASE	0.13–0.15	0.16–0.20	0.13–0.23	0.35–0.44
	SIPH / BL	0.03–0.04	0.02–0.03	0.02–0.03	0.02
	SIPH / ANT III	0.06–0.09	0.06–0.07	0.06–0.09	0.07–0.10
	SIPH / FEM	0.13–0.17	0.09–0.12	0.09–0.13	0.09–0.12
	SIPH / Cauda	0.57–0.79	0.27–0.36	0.36–0.64	0.20–0.35
	Ls ANT III / BD III	0.25–0.50	0.33	0.20–0.33	0.33–0.67

In total, 63 COI sequences of five *Takecallis* species were downloaded from GenBank (Suppl. material 1). All sequences were aligned using MEGA 7 (Kumar et al. 2016). Intra- and inter-specific distances were calculated by a pairwise distance method based on the Kimura-2-Parameter (K2P) model (Kimura 1980). A neighbor-joining analysis (NJ) based on the K2P model for the final data set of 658 bp was also constructed.

## Taxonomy

### Takecallis

Taxon classificationAnimaliaHemipteraAphididae

Mastumura, 1917

Takecallis Matsumura, 1917: 354, 373.

#### Type-species.

*Takecallis bambusae* Matsumura, 1917 (= *T. arundicolens*) by original designation.

#### Generic diagnosis.

Alatae: Morphological features of *Takecallis* are similar to *Subtakecallis* Raychaudhuri and Pal in having a nose-like processus on the clypeus, and spinal abdominal setae surrounded by cribriform wax glands. However, this genus can be distinguished from the above genus by the following characters: PT/BASE ≥ 1.00 and spinal abdominal setae often at low elevations. Apterae are unknown.

#### Host plant.

*Takecallis* species occur on various bamboos such as *Arundinaria* spp., *Bambusa* spp., *Dendrocalamus* spp., *Pseudosasa* spp., *Phyllostachys* spp., *Pleioblastus* spp., and *Sasa* spp. (Poaceae).

#### Distribution.

This genus is native to the Oriental region, but one or more species occur as introduced populations in Australian, Ethiopian, Palearctic, Nearctic, and Neotropical regions.

### Takecallis alba

Taxon classificationAnimaliaHemipteraAphididae

Y. Lee sp. n.

http://zoobank.org/974AEFD4-E563-4944-8C2B-3039BC4099E9

**Figure 1.**
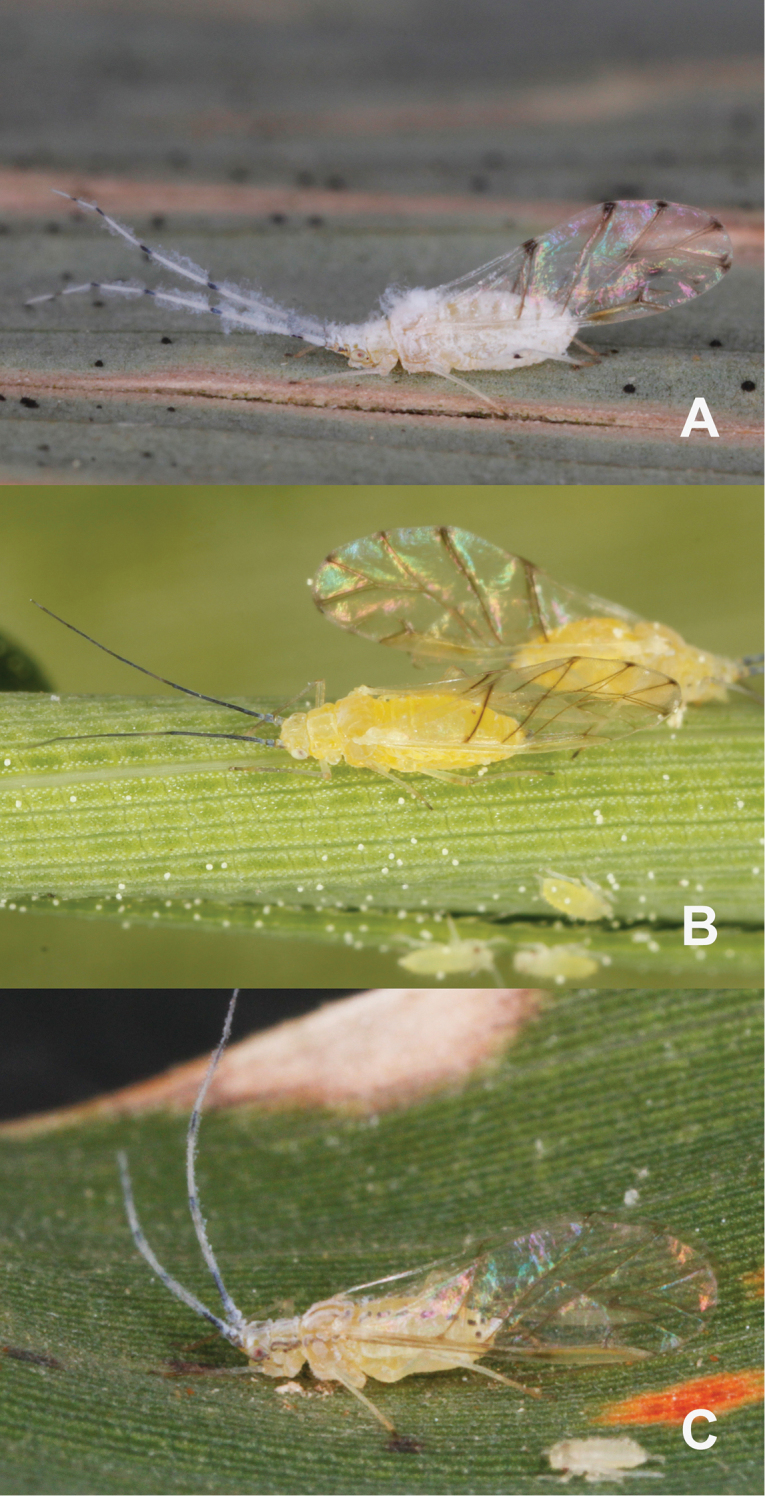
Photographs of live *Takecallis* spp. **A** alate viviparous female of *T. alba* sp. n. **B** alate viviparous female of *T. arundicolens* **C** alate viviparous female of *T. arundinariae*.

**Figure 2.**
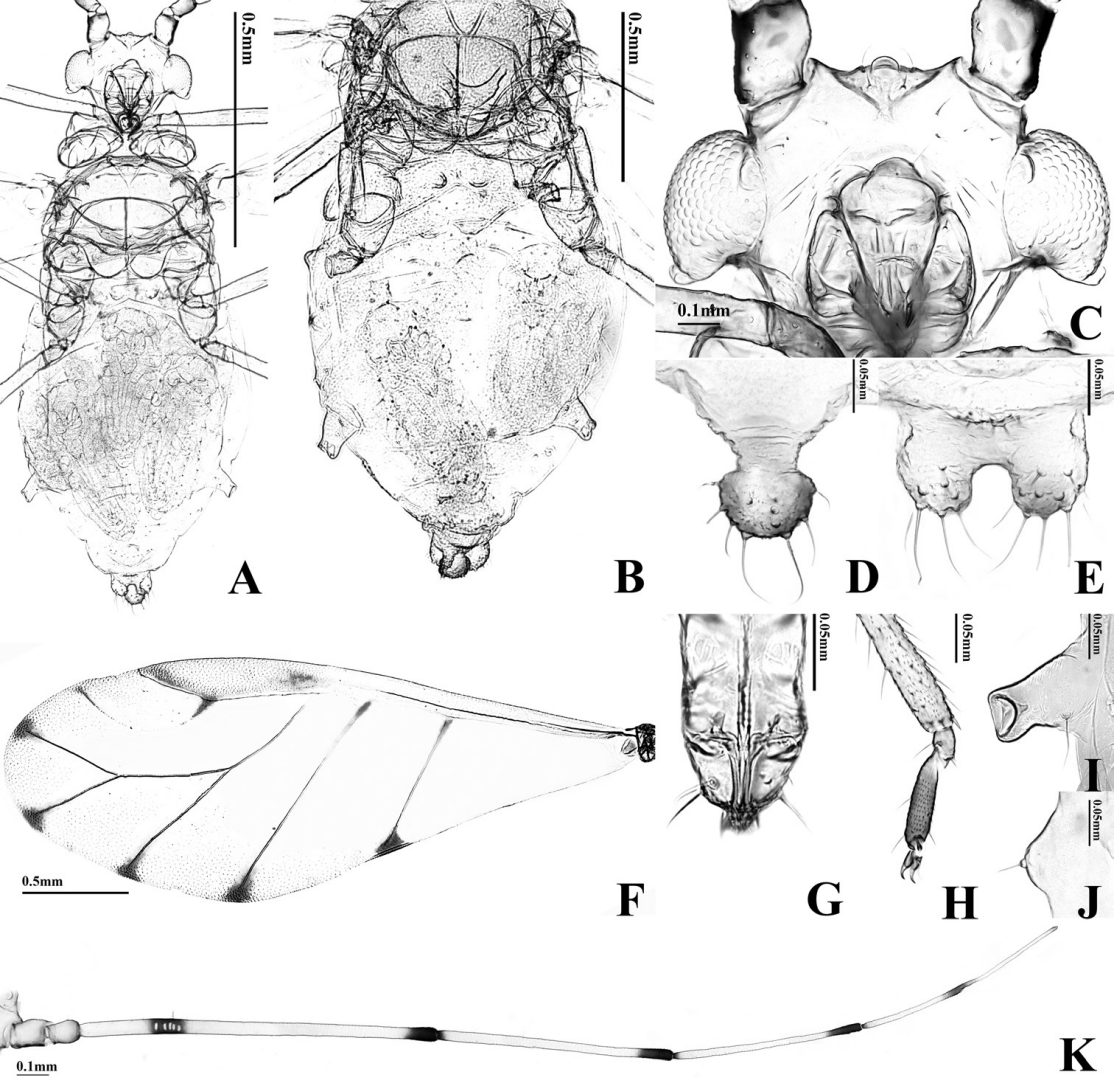
Alate viviparous female of *T. alba* sp. n.: **A** body **B** dorsal ABD TERG **C** head **D** cauda **E** anal plate **F** forewing **G** URS **H** HT 2 **I** SIPH **J** 4th marginal tubercle **K** ANT.

Figs 1A , 2 , Table 1

#### Material examined.

Holotype: 1 alate viviparous female, Mt. Hwangbyeong, Pyeongchang-gun, GW, South Korea, 37°42'27"N, 128°41'14"E, on *Sasa* sp., Y. Lee leg., 29.viii.2013, no. 130829YR-11; *Paratypes*: 10 alate viviparous females, same data as the holotype; 7 alate viviparous females, Mungyeong-eup, Mungyeong-si, GN, South Korea, 36°47'11"N, 128°09'29"E, on *Pseudosasa* sp., S. Lee leg., 18.v.2005, no. 050518SH-38; 6 alate viviparous females, Mt. Hwangbyeong, Pyeongchang-gun, GW, South Korea, 37°42'27"N, 128°41'14"E, on *Sasa* sp., Y. Lee leg., 15.viii.2013, no. 130815YR-12; 5 alate viviparous females, Mt. Deokyousan, Muju-gun, JB, South Korea, 35°54'23"N, 127°48'51"E, on *Pseudosasa* sp., H. Lee leg., 30.vi.2014, no. 140630YR-2; 2 alate viviparous females, Inje-gun, Hangye-ri, Hangyeryng, GW, South Korea, 38°6'31"N, 128°24'49"E, on *Sasa* sp., Y. Lee leg., 15.vi.2015, no. 150615YR-3.

#### Etymology.

The species name *alba* is derived from Latin, referring to its pale body color.

#### Diagnosis.

*T. alba* sp. n. is morphologically close to *T. assumenta* Qiao and Zhang and *T. affinis* Ghosh. However, this species can be distinguished from the latter two species by the following characters: URS with four accessory setae (accessory setae absent in *T. assumenta*, two accessory setae in *T. affinis*), URS 0.45–0.50 × HT 2 (0.43 in *T. assumenta*, 0.32–0.41 in *T. affinis*), ANT III with 4–7 transversely elliptical secondary rhinaria densely concentrated on very short dark section of proximal 3rd of ANT III (6–10 elliptical secondary rhinaria, on basal 1/3 of the segment in *T. assumenta*, 10–16 subcircular secondary rhinaria on basal 2/5 of the segment in *T. affinis*).

#### Description.

*Alate viviparous female*: *Color in life*. Head pale to yellow, compound eye red. ANT pale, marginal border of ANT I-II dusky, the top end of 1/3 of the segment, and distal joint of ANT III dark, distal joint of ANT IV - BASE dusky. Thorax and ABD TERG pale yellow to bright yellow. Legs pale, distal 2/5 of FEM with dark spot, tarsi dark. Wing veins dark, margins of wing veins with dark spots. SIPH pale. Cauda slightly dark. Entire body covered with white wax.

*Morphology*. Body oval, BL 2.08–2.51 mm long. Head with three pairs of anterior and two pairs of posterior short and pointed discal setae about 0.02–0.03 mm long, median protrusion on frons developed, epicranial suture and antennal tubercle developed, head dorsum without tubercles. ANT 6-segmented, 1.48–1.70 × BL, ANT III longest with 4–7 transversely elliptical secondary rhinaria in a row on the top end of 1/3 of the segment, Ls ANT III 0.25–0.50 × BD III, ANT IV-VI imbricated, ANT IV without secondary rhinaria, ANT IV longer than ANT V, PT 0.89–1.11 × BASE. Clypeus with nose-like processus bearing two hairs. Rostrum very short, barely reaching to fore coxae, URS short blunted, 0.05 mm long with four accessory setae, URS 0.13–0.15 × BASE, 0.45–0.50 × HT 2. Thorax smooth without tubercles. Fore coxae enlarged. Longest setae on TIB 0.06–1.00 × middle width of TIB, first tarsal segments with 6–7 setae, HT 2 0.10–0.11 mm long. Wing vein Pts of forewing slightly dark, margins of wing veins Cu1b, Cu1a, and M with dark spots. Dorsal ABD TERG I–VII with a pair of spinal setae on small elevations, ABD TERG VIII with a pair of spinal setae on a single elevation, ABD TERG margin I–IV with a single seta on cone-shaped marginal tubercle, 4th marginal tubercle 0.04–0.05mm. SIPH cylindrical 0.08–0.11 mm long, bearing 0.03–0.05 mm of single seta. Cauda knobbed 0.12–0.14 mm long with 9–12 setae. Anal plate bilobed, each lobe with 7–10 setae.

#### Distribution.

This species has so far been collected from Gyeongsangnam-do, Gangwon-do, and Jeollanam-do of South Korea.

#### Host plants.

This species feeds on the underside of leaves of *Pseudosasa* sp., and *Sasa* spp. (Poaceae). The host plants were identified by the first author using Lee (2003).

#### Remarks.

This species was first referred to as *Takecallis* sp. in Lee et al. 2017.

### Takecallis arundicolens

Taxon classificationAnimaliaHemipteraAphididae

(Clarke, 1903)

**Figure 3.**
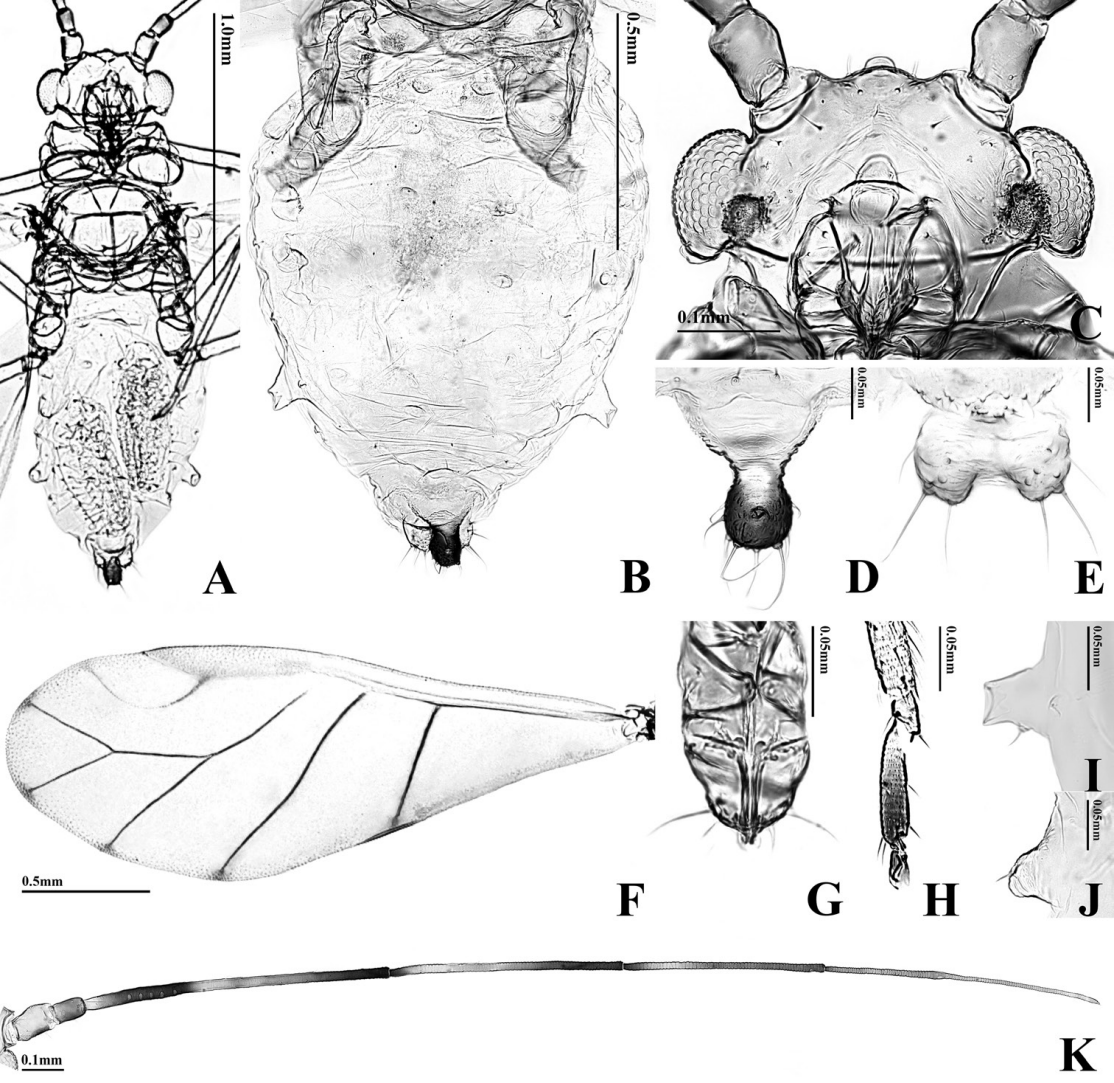
Alate viviparous female of *T. arundicolens*: **A** body **B** dorsal ABD TERG **C** head **D** cauda **E** anal plate **F** forewing **G** URS **H** HT 2 **I** SIPH **J** 4th marginal tubercle **K** ANT.

Figs 1B , 3 , Table 1

Takecallis bambusae Matsumura, 1917.

#### Material examined.

2 alate viviparous females, Naksan-temple, Ganghyeon-myeon, Yangyang-gun, GW, South Korea, 38°7'25"N, 128°37'38"E, on *Sasa* sp., S. Lee leg., 25.vi.2003, no. 030625SH-62; 1 alate viviparous female, Namheae-gun, GN, South Korea, 34°50'15"N 127°53'32"E, on *Sasa* sp., S. Lee leg., 7.iv.2006, no. 060407SH-16; 1 alate viviparous female, Seobjikoji Beach, Seoguipo-si, JJ, South Korea, 33°25'24"N, 126°55'45"E, on *Sasa* sp., S. Lee leg., 27.iv.2006, no. 060427SH-55; 3 alate viviparous females, Ehwa womans univ., Deahyeon-dong, Seodaemun-gu, Seoul, South Korea, 37°33'42"N, 126°56'48"E, on *Arundinaria* sp., Y. Lee leg., 18.x.2011, no. 111018YR-1; 2 alate viviparous females, Taean-gun, CN, South Korea, 36°47'47"N, 126°09'04"E, on *Sasa* sp., Y. Lee and H. Lee leg., 10.v.2014, no. 140510YR-17; 1 alate viviparous female, Is. Odongdo, Yeosu-si, JN, South Korea, 34°44'51"N, 127°45'52"E, on *Sasa* sp., Y. Lee and H. Lee leg., 16.vii.2014, no. 140716YR-1.

#### Description.

*Alate viviparous female*: *Color in life*. Head pale to bright yellow, compound eye pale. ANT I concolorous with head, ANT II slightly dusky, basal 1/3 and distal 1/3 of ANT III dark, basal half of ANT IV-V, and ANT VIb dusky. Thorax concolorous with head or slightly darker. ABD TERG pale yellow to bright yellow. Legs pale, tarsi dark. Wing veins dark. SIPH pale. Cauda dark. Entire body slightly covered with white wax.

*Morphology*. Body oval, BL 1.57–1.89 mm long. Head with 3 pairs of anterior and 2 pairs of posterior short and pointed discal setae about 0.02–0.04mm, median protrusion on frons developed, epicranial suture and antennal tubercle developed, head dorsum without tubercles. ANT 6-segmented, 1.30–1.60 × BL, ANT III longest with 4–7 transversely elliptical secondary rhinaria in a row on 1/3 of the segment, Ls ANT III 0.33 × BD III, ANT IV–VI imbricated, ANT IV without secondary rhinaria, BASE with a single seta, PT 1.10–1.26 × BASE. Clypeus with nose-like processus, rostrum very short, reaching to fore coxae, URS short blunted, 0.05–0.06 mm long with 4–5 accessory setae, URS 0.16–0.20 × BASE, 0.56–0.67 × HT 2. Thorax smooth and without tubercles. Fore coxae enlarged, longest setae on TIB almost same length as middle width of TIB, first tarsal segments with 5–7 setae, HT 2 0.09–0.10 mm long. Wing vein Pts of forewing slightly dark. Dorsal ABD TERG I–VII with a pair of spinal setae on small elevations, ABD TERG VIII with a pair of spinal setae, ABD TERG margin I-IV with a single seta on cone-shaped marginal tubercle, 4th marginal tubercle 0.04–0.05mm. SIPH cylindrical 0.04–0.05 mm long with 0.02–0.03 mm of single seta. Cauda knobbed 0.14–0.15 mm long with 9–13 setae. Anal plate bilobed, each lobe with 7–8 setae.

#### Distribution.

This species is originally distributed in East Asian countries; Korea (Paik 1965), China (Qiao and Zhang 2004), Japan (Higuchi 1968), and eastern Russia (Blackman and Eastop 2017). It has been introduced into Europe (Pons and Lumbierres 2004, Stroyan 1964), and USA (California) (Clarke 1903). However, the recent DNA barcoding result revealed that European populations are genetically different from Asian populations (Lee et al. 2017).

#### Host plants.

*Arundinaria* spp., *Bambusa* spp., *Phyllostachys* spp., and *Sasa* spp. (Poaceae).

### Takecallis arundinariae

Taxon classificationAnimaliaHemipteraAphididae

(Essig, 1917)

**Figure 4.**
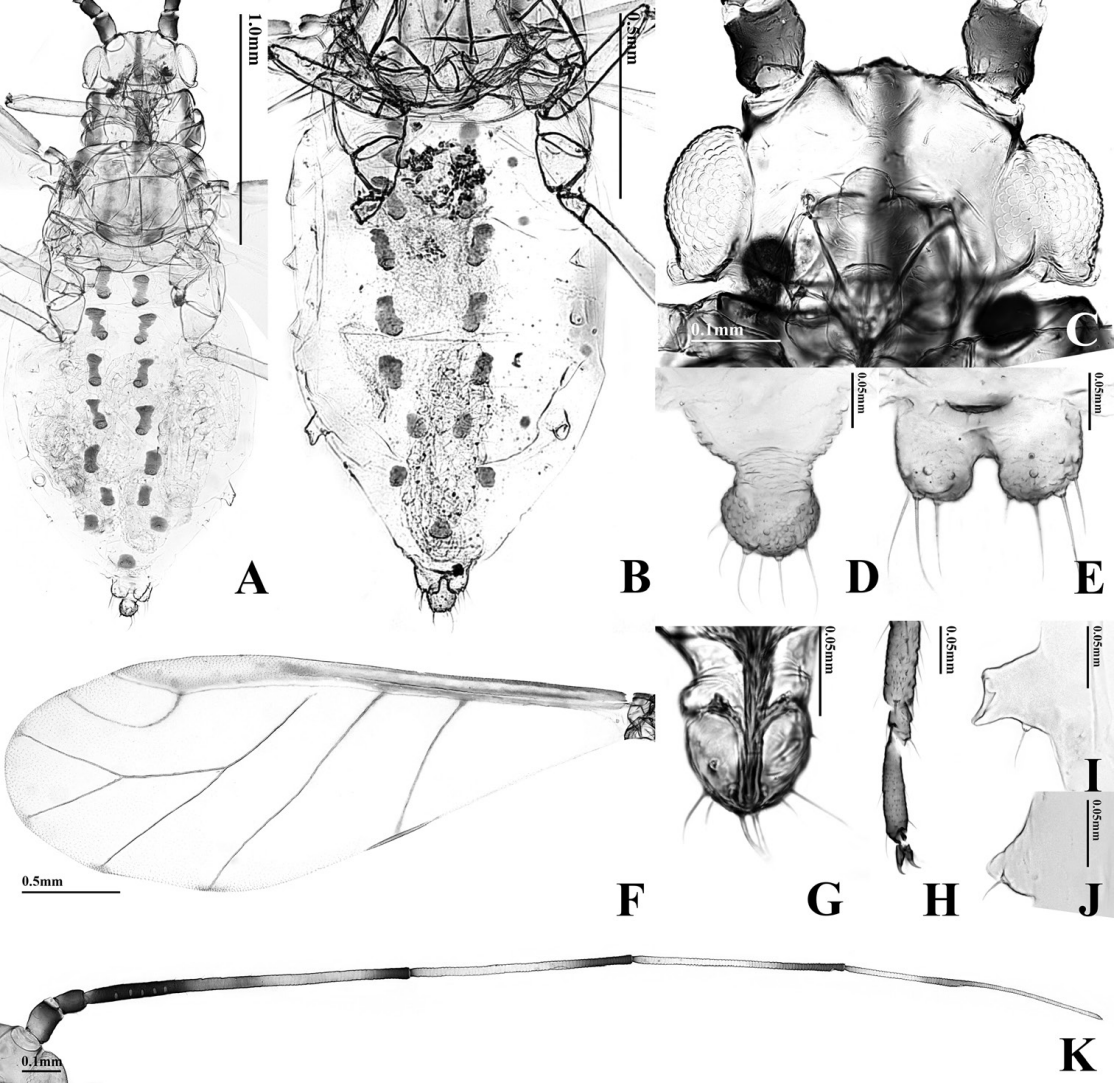
Alate viviparous female of *T. arundinariae*: **A** body **B** dorsal ABD TERG **C** head **D** cauda **E** anal plate **F** forewing **G** URS **H** HT 2 **I** SIPH **J** 4th marginal tubercle **K** ANT.

Figs 1C , 4 , Table 1

Takecallis arundinariae Blackman, 1980.
Myzocallis bambucifoliae Takahashi, 1921.
Myzocallis bambusifoliae Takahashi, 1921.

#### Material examined.

1 alate viviparous female, Seoul, South Korea, 37°34'31"N, 126°59'51"E, on *Sinoarundinaria reticulata*, W.H. Paik leg., 15.v.1960, no. 1258; 1 alate viviparous female, Seoul, South Korea, 37°34'31"N, 126°59'51"E, on *Sasa kurilensis*, W.H. Paik leg., 3.xi.1971, no. 6924; 5 alate viviparous females, Hwasun, JN, South Korea, 35°3'52"N, 126°59'11"E, on unknown host, S. Lee leg., 31.iii.1999, no. 990331SH-1; 6 alate viviparous females, Chupungryeong, Gimcheon, GB, South Korea, 36°13'9"N, 127°59'51"E, on *Sasa* sp., S. Lee leg., 12.v.1999, no. 990512SH-30; 5 alate viviparous females, Sanpo-myeon, Naju-si, JN, South Korea, 35°2'22"N, 126°48'21"E, on *Phyllostachys bambusoidea*, G.M. Kwon leg., 12.i.2000, no. 000112GM-04; 5 alate viviparous females, Namyang-myeon, Goheung-gun, JN, South Korea, 34°43'42"N, 127°20'10"E, on *Phyllostachys bambusoidea*, S. Lee leg., 14.iii.2000, no. 000314SH-2; 3 alate viviparous females, Sacheon-gun, GN, South Korea, 37°48'39"N, 128°51'17"E, on *Phyllostachys bambusoidea*, S. Lee leg., 16.iii.2000, no. 000316SH-6; 4 alate viviparous females, Namhae-gun, GN, South Korea, 34°49'58"N, 127°53'53"E, on *Gramineae* sp., S. Lee leg., 8.iv.2006, no. 060408SH16; 2 alate viviparous females, Taean-gun, CN, South Korea, 36°44'44"N 126°17'52"E, on *Phyllostachys* sp., Y. Lee and H. Lee leg., 10.v.2014, no. 140510YR-17.

#### Description.

*Alate viviparous female*: *Color in life*. Head pale to bright yellow with black stripe on head dorsum, compound eye pale red. From ANT I to basal half of ANT III dark, from distal joint of ANT III to BASE dusky. Thorax pale yellow with dark stripe pattern. ABD TERG pale yellow with pair of dark dorsal tubercle. Legs pale, tarsi dark. Wing veins dark. SIPH and cauda pale. Entire body slightly covered with white wax.

*Morphology*. Body oval, BL 1.90–2.65 mm long. Head with three pairs of anterior and two pairs of posterior short and pointed discal setae about 0.010.02mm, median protrusion on frons developed, epicranial suture and antennal tubercle developed, head dorsum without tubercles. ANT 6-segmented, 1.22–1.39 × BL, ANT III longest with 5–10 transversely elliptical secondary rhinaria in a row on 1/4 of the segment, Ls ANT III 0.20–0.33 times as long as BD III, ANT IV-VI imbricated, ANT IV without secondary rhinaria, PT 0.94–1.27 times as long as BASE. Clypeus with nose-like processus, rostrum very short, passing over fore coxae, URS short blunted, 0.050.06 mm with four accessory setae, URS 0.13–0.23 × BASE, 0.42–0.60 × HT 2. Thorax smooth and without tubercles. Fore coxae enlarged, longest setae on TIB 0.75–1.25 × middle width of TIB, first tarsal segments with 5–7 setae, HT 2 0.10–0.12 mm long. Wing veins Co and Pts of forewing slightly dark. Dorsal ABD TERG I–VII with a pair of spinal setae on small elevations, ABD TERG VIII with a pair of spinal setae. SIPH cylindrical, 0.05–0.07 mm long bearing about 0.03–0.06 mm of single seta. Cauda knobbed 0.11–0.16 mm long with 10–15 setae. Anal plate bilobed, each lobe with 8–12 setae.

#### Distribution.

This species is originally distributed in south-east Asian countries; Korea (Paik 1965), China (Qiao and Zhang 2004), India (Gosh et al. 1971), Japan (Higuchu 1968), Taiwan (Higuchu 1968), and eastern Russia (Blackman and Eastop 2017). It has invaded Europe (Barbagallo and Ortu 2009, Basky and Neményi 2014, Giacalone and Lampel 1996, Higuchi 1968, Limonta 1990, Piron 2009, Tistispis et al. 2007), Australia (Valenzuela et al. 2010), New Zealand (Blackman and Eastop 2017), North America (Coﬀelt and Schultz 1990), and South America (Foureaux and Kato 1999, Lazzari et al. 1999, Simbaqueba et al. 2016).

#### Host plants.

*Arundinaria* spp., *Bambusa* spp., *Dendrocalamus* spp., *Phyllostachys* spp., *Sasa* spp., and *Sinoarundinaria reticulata* (Poaceae).

#### Remarks.

Among the examined specimens, it is described that four alate viviparous females were collected on *Gramineae* sp. However, this host plant is probably not a true host plant due to *Takecallis* species being recorded only on bamboo species, and is probably a misidentification.

### Takecallis taiwana

Taxon classificationAnimaliaHemipteraAphididae

(Takahashi, 1926)

Fig. 5 , Table 1

Therioaphis tectae Tissot, 1932.

#### Material examined.

6 alate viviparous females, Seoguipo-si, JJ, South Korea, 33°15'3"N, 126°32'38"E, on *Sasa* sp., W.H. Paik leg., 25.iv.1971, no. 6196; 8 alate viviparous females, Seoguipo-si, JJ, South Korea, 33°15'3"N, 126°32'38"E, on *Sasa* sp., W.H. Paik leg., 15.x.1971, no. 6799.

#### Description.

*Alate viviparous female*: *Color in life*. Not available in this study.

*Morphology*. Body oval, BL 2.21–2.48 mm long. Head with three pairs of anterior and two pairs of posterior pointed discal setae about 0.04–0.05mm, median protrusion on frons developed, epicranial suture and antennal tubercle developed, head dorsum with a central black stripe, spinal tubercle not developed. ANT 6-segmented 0.73–0.79 × BL, ANT III longest with 5–7 transversely elliptical secondary rhinaria in a row on basal 1/3 of the segment, longest setae on ANT III 0.33–0.67 × BD III, from distal half of ANT III to ANT VI imbricated, ANT IV without secondary rhinaria, PT 0.901.06 × BASE. Clypeus with nose-like processus, rostrum very short, reaching to fore coxae, URS short blunted 0.07 mm long with 4–5 accessory setae, URS × 0.35–0.44 BASE, 0.54–0.64 × HT 2. Thorax smooth, without tubercles. Fore coxae weakly enlarged, longest setae on TIB 0.08–1.00 × middle width of TIB, first tarsal segments with 5–7 setae, HT 2 0.11–0.13 mm long. Wing veins Co and Pts of forewing slightly dark. Dorsal ABD TERG I–VII with a pair of spinal setae on small elevations, ABD TERG VIII with 2 setae. SIPH cylindrical, 0.04–0.06 mm long. Cauda knobbed 0.15–0.20 mm long with 12–13 setae. Anal plate bilobed, each lobe with ten setae.

**Figure 5.**
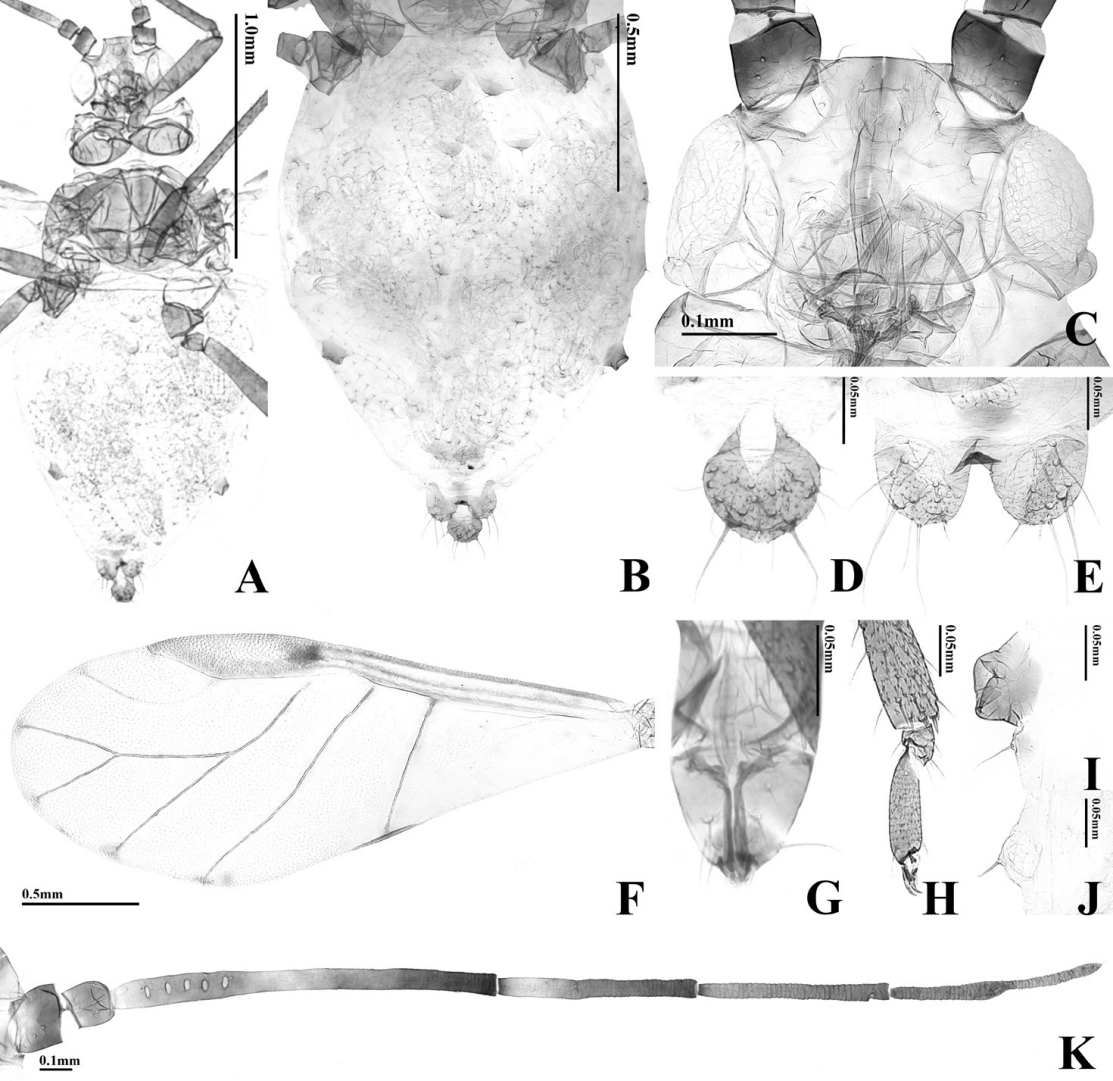
Alate viviparous female of *T. taiwana*: **A** body **B** dorsal ABD TERG **C** head **D** cauda **E** anal plate **F** forewing **G** URS **H** HT 2 **I** SIPH **J** 4th marginal tubercle **K** ANT.

#### Distribution.

This species is widely distributed in Southeast Asia; Korea (Paik 1965), China (Qiao and Zhang 2004), Japan (Higuchi 1968), and Taiwan (Higuchi 1968). It has been introduced into Europe (Higuchi 1968, Maslyakov and Izhevsky 2011, Ripka 2008, Simala et al. 2008), South Africa (Quednau 1962), New Zealand (Blackman and Eastop 2017), North America (Halbert et al. 2000), and South America (Foureaux and Kato 1999, Lazzari et al. 1999).

#### Host plants.

*Arundinaria* spp., *Bambusa* spp., *Phyllostachys* spp., and *Sasa* spp. (Poaceae).

#### Remarks.

This species was misidentified as *T. sasae* by Paik (1972) in Korea. Later it was revised to *T. taiwana* by Quednau and Lee (2001).

**Figure 6.**
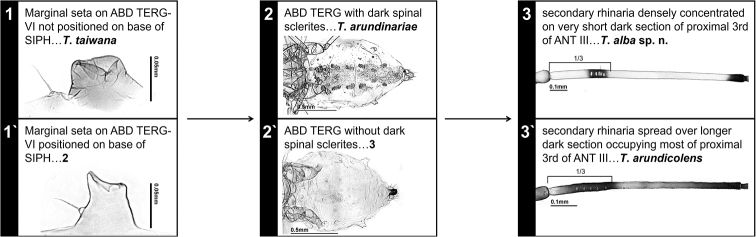
Pictorial key to species of the genus *Takecallis* in Korea.

##### Key to species of the genus *Takecallis* in Korea (Fig. 6)

**Table:** 

1	ANT shorter than BL, marginal seta on ABD TERG VI not positioned on base of SIPH (Fig. 5I), URS 0.35–0.44 × BASE	***T. taiwana***
–	ANT longer than BL, marginal seta on ABD TERG VI positioned on base of SIPH (Figs 2I, 3I, 4I), URS 0.13–0.23 × BASE	**2**
2	ABD TERG with dark spinal sclerites (Fig. 4B), cauda pale (Fig. 4D)	***T. arundinariae***
–	ABD TERG without dark spinal sclerites (Figs 2B, 3B), cauda slightly dusky or blackish (Figs 2D, 3D)	**3**
3	ANT 3.36–4.00mm, secondary rhinaria densely concentrated on very short dark section of proximal third of ANT III (Fig. 2K)	***T. alba* sp. n.**
–	ANT 2.36–2.51mm, secondary rhinaria spread over longer dark section occupying most of proximal third of ANT III (Fig. 3K)	***T. arundicolens***

##### Molecular analyses and discussion

**Figure 7.**
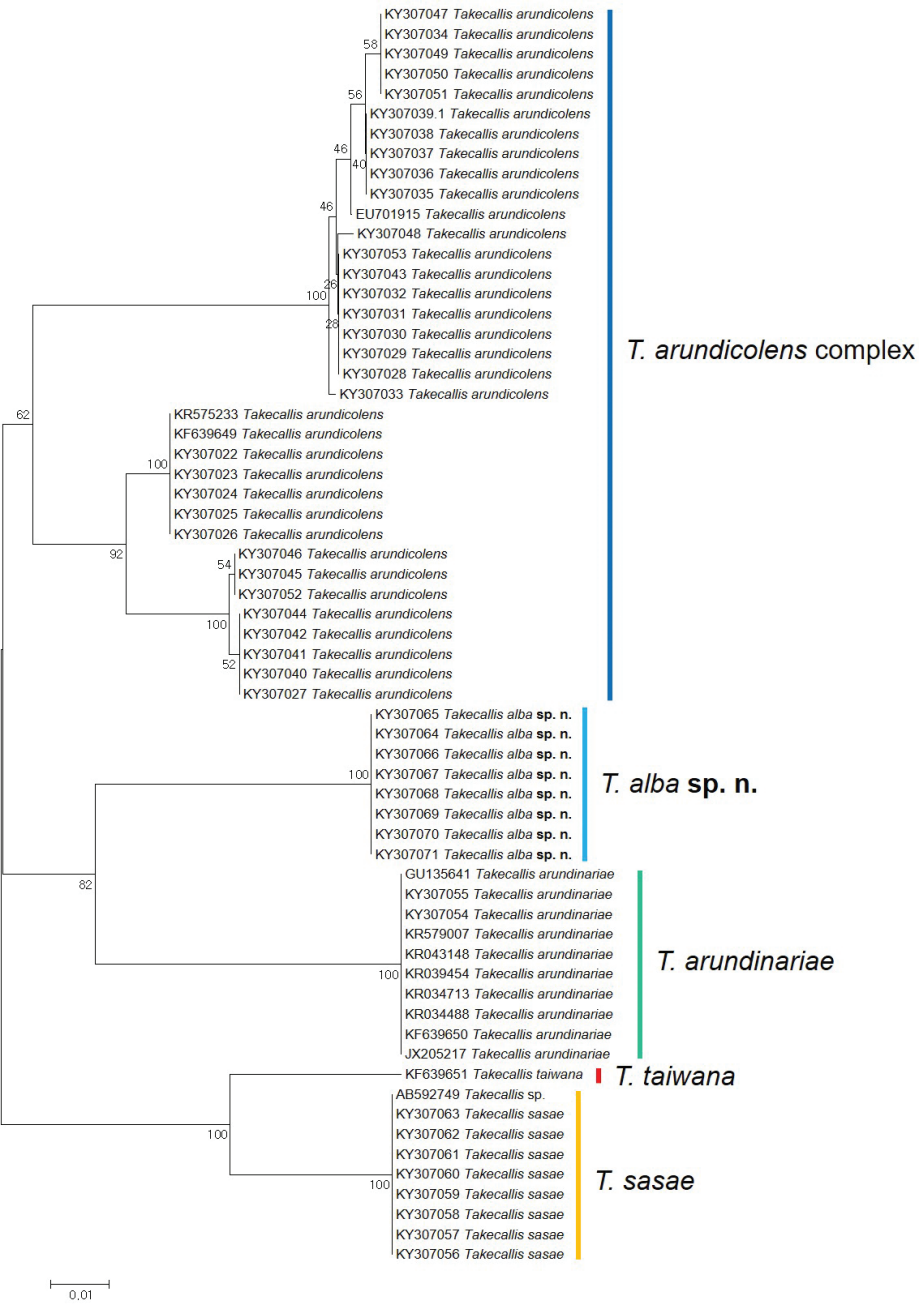
Neighbor-joining tree of COI partial gene sequences of *Takecallis* spp. (63 sequences of five species).

The NJ tree of partial COI sequences suggested that 63 sequences are distinctly divided into six groups (Fig. 7). This result clearly represented each morpho-specific group except the *T. arundicolens* complex. The *T. arundicolens* complex was separated into two genetically distinct groups (Fig. 7). Genetic distances between the two *T. arundicolens* groups ranged from 7.16 % to 9.36 %. These intraspecific divergence values are much higher than the general species delimitation value of 2.5 % in the subfamily Calaphidinae (Lee et al. 2017). In the previous study, Lee et al. (2017) suggested that this species complex seems to include at least 2 distinct species. However, it is very difficult to determine which one is the original species because morphological differences between genetically distinct groups were only observed in alatoid nymphs (Lee et al. 2017). Therefore, to solve this issue explicitly, additional studies are needed.

Except for the *T. arundicolens* complex, the rest of the four species showed 0 % of intraspecific genetic divergence (Table 2). Interspecific distances among the five species ranged from 5.71 % to 14.44 % (Table 2). *T. sasae* and *T. taiwana* showed the lowest interspecific distance level (Table 2). Overall mean genetic distance was 8.91 % for the 63 partial COI sequences of the five *Takecallis* species.

**Table 2.** Intra- and inter-specific pairwise genetic divergence (%) based on K2P model for five *Takecallis* species

	*T. alba* sp. n.(n = 8)	*T. arundicolens* (n = 35)	*T. arundinariae* (n = 11)	*T. sasae* (n = 8)	*T. taiwana* (n = 1)
*T. alba* sp. n.	0				
*T. arundicolens*	9.36–12.58	0–9.36			
*T. arundinariae*	9.94	7.78–12.87	0		
*T. sasae*	13.46	10.51–12.49	14.44	0	
*T. taiwana*	11.14	9.58–13.50	14.44	5.71	0

Molecular evidence strongly indicates the validity of *T. alba* sp. n. All of the individuals of *T. alba* sp. n. were grouped together and this group was clearly separated from other species groups with a high interspecific distance level that ranged from 9.36 % to 13.46 % (Table 2). Morphological characteristics of this species correspond to molecular evidence. Although we could not test all *Takecallis* species from all over the world, this species also has morphological characteristics that distinguish it from all known species. Morphologically, *T. alba* sp. n. is most similar to *T. affinis* and *T. assumenta*. However, its number of accessory setae on URS and the arrangement of secondary rhinaria on ANT III are clearly distinct from the above two species.

In the present study, four *Takecallis* species were recognized from Korea. Our study demonstrated that the four species are clearly separated based on morphological and molecular evidence. However, the taxonomic status of genetically distinct groups within the *T. arundicolens* complex still needs to be resolved.

## Supplementary Material

XML Treatment for Takecallis

XML Treatment for Takecallis alba

XML Treatment for Takecallis arundicolens

XML Treatment for Takecallis arundinariae

XML Treatment for Takecallis taiwana

## Supplementary materials

Supplementary material 1 Detailed collection information and Genbank accession numbers Data type: molecular data This is an open access article distributed under the terms of the Creative Commons Attribution License (CC BY 4.0), which permits unrestricted use, distribution, and reproduction in any medium, provided the original author and source are credited.Yerim Lee, Seunghwan Lee
